# MicroRNAs and new biotechnological tools for its modulation and improving stress tolerance in plants

**DOI:** 10.1111/pbi.13116

**Published:** 2019-06-14

**Authors:** Marcos Fernando Basso, Paulo Cavalcanti Gomes Ferreira, Adilson Kenji Kobayashi, Frank G. Harmon, Alexandre Lima Nepomuceno, Hugo Bruno Correa Molinari, Maria Fatima Grossi‐de‐Sa

**Affiliations:** ^1^ Embrapa Genetic Resources and Biotechnology Brasília DF Brazil; ^2^ Federal University of Rio de Janeiro Rio de Janeiro RJ Brazil; ^3^ Embrapa Agroenergy Brasília DF Brazil; ^4^ Plant Gene Expression Center USDA‐ARS Albany CA USA; ^5^ Department of Plant and Microbial Biology UC Berkeley Berkeley CA USA; ^6^ Embrapa Soybean Londrina PR Brazil; ^7^ Post‐Graduation Program in Genomic Sciences and Biotechnology Catholic University of Brasília Brasília DF Brazil; ^8^ Post‐Graduation Program in Biotechnology Potiguar University (UNP) Natal RN Brazil

**Keywords:** plant molecular biology, genetic engineering tools, small interfering RNA, MIR genes, posttranscriptional gene silencing, miRNA modulation

## Abstract

MicroRNAs (miRNAs) modulate the abundance and spatial–temporal accumulation of target mRNAs and indirectly regulate several plant processes. Transcriptional regulation of the genes encoding miRNAs (MIR genes) can be activated by numerous transcription factors, which themselves are regulated by other miRNAs. Fine‐tuning of MIR genes or miRNAs is a powerful biotechnological strategy to improve tolerance to abiotic or biotic stresses in crops of economic importance. Current approaches for miRNA fine‐tuning are based on the down‐ or up‐regulation of MIR gene transcription and the use of genetic engineering tools to manipulate the final concentration of these miRNAs in the cytoplasm. Transgenesis, cisgenesis, intragenesis, artificial MIR genes, endogenous and artificial target mimicry, MIR genes editing using Meganucleases, ZNF proteins, TALENs and CRISPR/Cas9 or CRISPR/Cpf1, CRISPR/dCas9 or dCpf1, CRISPR13a, topical delivery of miRNAs and epigenetic memory have been successfully explored to MIR gene or miRNA modulation and improve agronomic traits in several model or crop plants. However, advantages and drawbacks of each of these new biotechnological tools (NBTs) are still not well understood. In this review, we provide a brief overview of the biogenesis and role of miRNAs in response to abiotic or biotic stresses, we present critically the main NBTs used for the manipulation of MIR genes and miRNAs, we show current efforts and findings with the MIR genes and miRNAs modulation in plants, and we summarize the advantages and drawbacks of these NBTs and provide some alternatives to overcome. Finally, challenges and future perspectives to miRNA modulating in important crops are also discussed.

## Background

Plants are constantly challenged by numerous adverse conditions that modulate their evolution (Crisp *et al*., 2016). Plant responses to stresses involve a broad regulation of numerous genes, interfering with several agronomic traits, such as plant growth and productivity (Hackenberg *et al*., 2015; Yi *et al*., 2015). Understanding these mechanisms is important for the development of biotechnological tools to improve desirable agronomic traits (Teotia *et al*., 2016; Zhang, 2015).

MicroRNAs (miRNAs) are short (21–24 nucleotide) RNAs that modulate the amount and spatial–temporal accumulation of target mRNAs and indirectly interfere with several plant pathways. These molecules are derived from noncoding RNAs arising from the gene expression of miRNAs (MIR genes). MIR gene transcription can be activated by numerous transcription factors that can be regulated by another set of miRNAs. In addition, MIR genes or their transcripts are often regulated by other miRNAs.

Several types of abiotic or biotic stresses alter MIR gene expression profiles, modulating the accumulation of miRNAs and consequently of the targeted mRNAs (Ferdous *et al*., 2015; Hackenberg *et al*., 2015). In plants, miRNA targets form a broad group of genes involved in numerous biological processes, including development and defence responses to pathogens, insects and environmental stresses (Hackenberg *et al*., 2015; Yi *et al*., 2015). The modulation of target gene expression by miRNAs may occur at the transcriptional (site‐specific DNA methylation mediated by miRNAs) and posttranscriptional (by mRNA degradation, translational inhibition or RNA deadenylation) levels (Borges and Martienssen, 2015). Stress‐associated regulatory networks involving the activity of miRNAs are poorly understood, and unravelling such mechanisms is further complicated by the fact that one miRNA may regulate several genes and that some genes may be regulated by multiple miRNAs.

The understanding of the complexity of MIR genes has rapidly increased as a consequence of next‐generation sequencing (NGS) and degradome sequencing technologies, as well as the exploitation of bioinformatics tools, open‐source miRNA databases and stem‐loop real‐time RT‐PCR. The constitutive, tissue‐specific, or stress‐ or senescence‐induced overexpression of specific MIR genes has been shown to improve desired agronomic traits in different plant species (Dong and Pei, 2014; Hajyzadeh *et al*., 2015; Ni *et al*., 2012; Trumbo *et al*., 2015; Zhang, 2015). In addition, artificial MIR genes, endogenous and artificial target mimicry, Meganucleases, ZNFs, TALENs, CRISPR/Cas9, CRISPR/Cpf1 or CRISPR/Cas13a systems, and pri‐miRNA or mature miRNA topical delivery have been shown to be useful for modulating miRNA accumulation.

## Plant small RNA biogenesis: a brief overview

RNA interference (RNAi) in plants is a well‐known mechanism that controls genome stability and epigenetic reprogramming, plant development, reproduction, defence responses and several other biological processes by regulating gene expression. Small interfering RNAs (siRNA) and miRNA are the two main small RNA (sRNA) classes involved in this regulation. The sRNAs are derived from single‐ or double‐strand RNA (ss‐ or dsRNA) intermediates that form hairpin‐like precursors and are subsequently processed by four DICER‐LIKE proteins (DCL1 to 4; reviewed by Borges and Martienssen, 2015).

**Figure 1:**
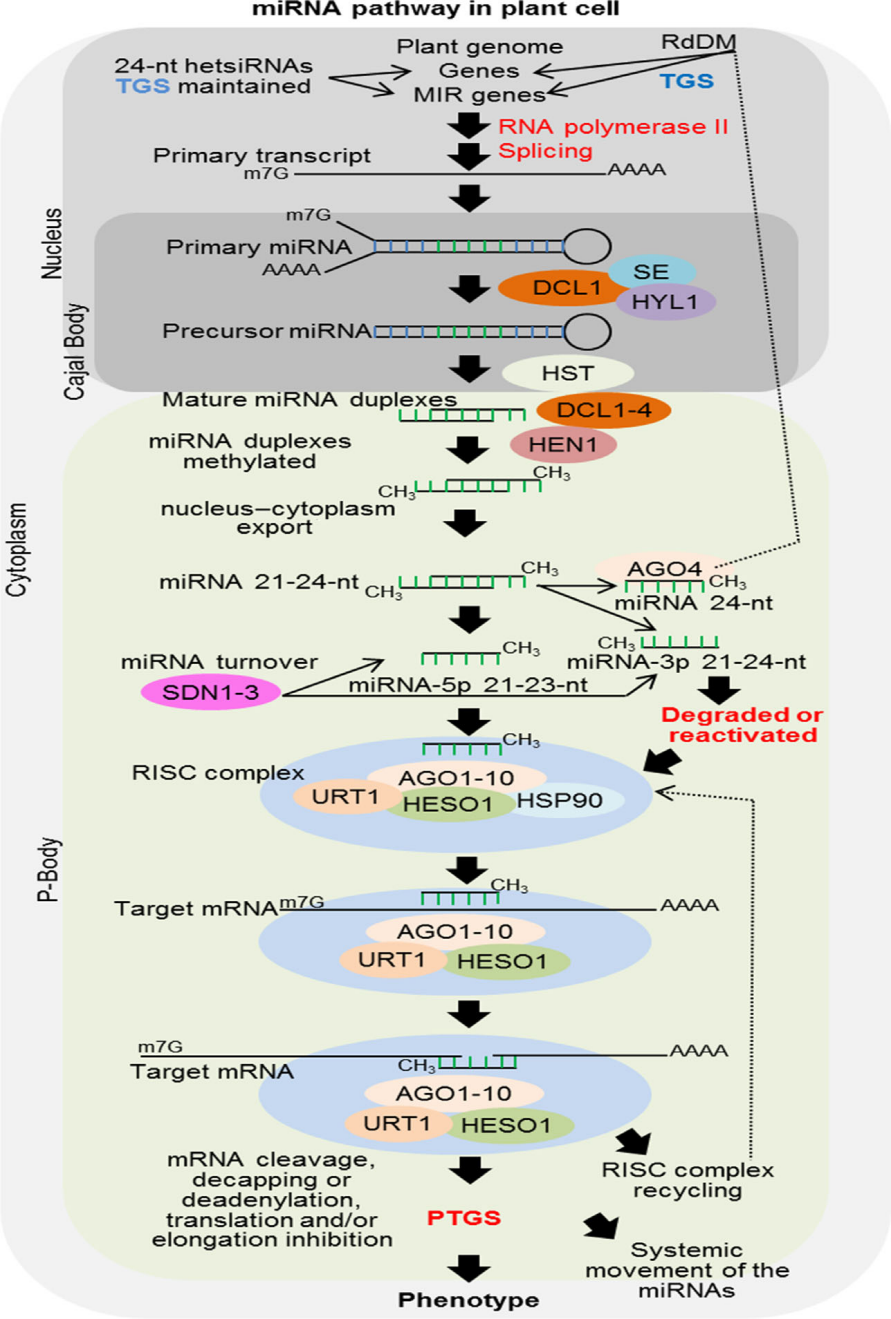
Overview of the miRNA pathway in *Arabidopsis thaliana*. In brief, MIR genes are transcribed by RNA polymerase II in the nucleus, and their primary transcripts are processed by the addition at 5′ terminus of a 7‐methyl guanosine cap, 3′ polyadenylated and RNA spliced. Then, the transcripts fold into primary miRNAs (pri‐miRNAs), which are quickly processed by DICER‐LIKE 1 (DCL1), supported by zinc‐finger protein SERRATE (SE), a dsRNA‐binding protein that interacts with DCL1 (HYL1) and other molecular factors, generating the precursor miRNAs (pre‐miRNAs) in Cajal bodies. Then, pre‐miRNAs are nucleus–cytoplasm transported by HASTY (HST) to P‐bodies. Next, the pre‐miRNAs are processed by DCL1 to DCL4 originating mature miRNA duplexes of 21–24 nucleotides (nt) in length, which are immediately methylated by HUA ENHANCER1 (HEN1) at the 3′ terminus. The double‐strand miRNA is separated, and single‐strand mature miRNA is integrated into RNA‐induced silencing complex (RISC), while the strand complementary to the mature miRNA (miRNA star) is degraded or “reactivated” and integrated into the RISC. Mature miRNAs of 21–23 nt in length associated with HEN SUPPRESSOR 1 (HESO1), UTP:RNA uridylyltransferase 1 (URT1), Heat Shock Protein 90 (HSP90), Argonaute proteins 1 to 10 (AGO1 to 10) and other molecular factors are integrated into the RISC, which successively scans all cytoplasmic messenger RNAs (mRNA) mainly based on sequence homology. MiRNAs act in posttranscriptional gene silencing (PTGS) by mediating target mRNA cleavage, decapping or deadenylation by AGO1 to 10 proteins or engage in translation and elongation repression. Additionally, miRNAs of 24 nt in length are complexed with AGO4 and directed to the nucleus, acting on RNA‐directed DNA methylation (RdDM), subsequently inducing transcriptional gene silencing (TGS). Thus, MIR genes can undergo TGS driven mainly by 24‐nt miRNAs, and this methylation is stabilized and maintained by heterochromatic siRNAs (hetsiRNAs). SMALL RNA DEGRADING NUCLEASE 1 to 3 (SDN1 to 3) functions in the turnover of miRNA‐5p or miRNA‐3p before it is directed to the RISC. Finally, miRNAs produced and accumulated in single cells can be systematically moved (cell‐to‐cell) by the vascular system comprising phloem and xylem and spreading to adjacent cells (reviewed by Borges and Martienssen, 2015).

miRNAs originate from species‐ or family‐specific noncoding RNAs transcribed from introns, exons or intergenic regions. MIR genes are generally transcribed by RNA polymerase II, originating the primary miRNA transcripts (pri‐miRNAs) that contain imperfect and self‐complementary foldback regions. Following 5′ m^7^G‐cap addition and 3′ polyadenylation, pri‐miRNAs are converted into miRNA precursor sequences (pre‐miRNAs) by DCL1 and other associated proteins (Borges and Martienssen, 2015). Pre‐miRNAs are exported from nuclear Cajal bodies to cytoplasmatic P‐bodies by HASTY protein and then processed by DCL1‐4 to generate miRNA duplexes of typically 21–24 nucleotides in length. Different DCLs can process a single pre‐miRNA, producing miRNA molecules of distinct sizes, for example, DLC1 and DCL4 of 21 nucleotides, DCL2 of 22 nucleotides and DCL3 of 24 nucleotides in length. These duplex miRNAs are 2′‐O‐methylated at both 3′‐ends by HUA ENHANCER 1 (HEN1), protecting the miRNAs from the uridylation and degradation initiated by the nucleotidyl transferases HEN1 SUPPRESSOR 1 (HESO1) and UTP:RNA uridylyltransferase 1 (URT1) (Tu *et al*., 2015). Then, mature miRNAs (miRNA‐5p or miRNA‐3p strand) are loaded onto Argonaute (AGO) proteins and incorporated into a RNA‐induced silencing complex (RISC), while miRNA star (miRNA*) molecules are most often degraded, although under certain circumstances these products might also be loaded onto AGO and complexed into a RISC. The miRNA‐associated AGO scans RNA molecules for sequence or near‐perfect sequence complementarity and promotes posttranscriptional gene silencing (PTGS) by catalysing the endonucleolytic cleavage, translation inhibition or deadenylation of the RNA target. Furthermore, Arabidopsis 24‐nucleotide miRNAs associate with AGO4, AGO6 and AGO9 to mediate the transcriptional gene silencing (TGS) of target genes through RNA‐directed DNA methylation (RdDM) (Borges and Martienssen, 2015; Figure 1). In addition, miRNA compartmentalization has been described in plants. For example, in Arabidopsis, miR390, AGO7 and TAS3 transcripts are complexed into cytoplasmic siRNA bodies that function in the biogenesis of TAS3‐derived trans‐acting siRNA (Martínez de Alba *et al*., 2015).

Posttranscriptional gene silencing can be amplified when target RNAs are cleaved and recognized by RNA‐dependent RNA polymerase (RDR) proteins, resulting in the *de novo* formation of dsRNA molecules, which are processed by DCLs into secondary sRNAs. These secondary sRNAs accumulate in the cytoplasm and can move cell‐to‐cell through plasmodesmata, constituting the systemic silencing signal (Borges and Martienssen, 2015).

## The role of miRNA in plant abiotic or biotic stress responses

Abiotic stresses, such as water deficit, salinity, low temperature, high temperature, heavy metal exposure, nutritional deprivation and high light intensity, as well as biotic stresses, such as viruses, bacteria, fungi, nematodes and insects, are major constraints to crop production worldwide. Throughout their evolution, plants have improved and developed mechanisms to respond and adapt to stressful conditions, including pathways in which miRNAs play a critical role in promoting stress tolerance (Shriram *et al*., 2016). MIR genes are up‐ or down‐regulated in response to abiotic (reviewed by Ferdous *et al*., 2015; Hackenberg *et al*., 2015) and biotic stresses (Gupta *et al*., 2014) in numerous species, including soya bean, sugarcane, rice, maize, wheat and tomato. Studies on the expression or accumulation of these miRNAs have provided several lines of evidence to better understand the regulatory networks associated with defence mechanisms against different types of stresses. From these findings, several biotechnological tools have been applied for fine‐tuning these networks and improving tolerance to stresses in important crops (Table 1).

**Table 1:** Plants genetically engineered to improve tolerance to biotic or abiotic stresses using constitutive overexpression of canonical or artificial miRNAs (amiRNAs), short tandem target mimicry (STTM) and synthetic mRNAs resistant to specific miRNAs

miRNA source	MIR gene or miRNA	Expression control	Transgenic plants	Phenotype in transgenic plants	References
*Arabidopsis thaliana* and *Lactuca sativa*	Ath‐miR156 and Ls‐miR156	*Cauliflower mosaic virus* (CaMV) 35S promoter	*Arabidopsis thaliana*	Flowering times were significantly delayed	Huo *et al*. (2016)
*Arabidopsis thaliana*	Ath‐miR156 and its target mimicry (STTM156)	CaMV 35S promoter	*Arabidopsis thaliana*	Improves stress tolerance, whereas MIM156 increased susceptibility	Cui *et al*. (2014)
*Brassica campestris*	Bra‐miR158a2	CaMV 35S promoter	*Brassica campestris*	Several pleiotropic phenotypes	Ma *et al*. (2016)
*Oryza sativa*	Os‐miR160a and Os‐miR398b	CaMV 35S promoter	*Oryza sativa*	Resistance to *Magnaporthe oryzae*	Li *et al*. (2014)
*Arabidopsis thaliana*	Ath‐miR164	–	*Arabidopsis thaliana*	Alters the leaf longevity	Kim *et al*. (2009)
*Arabidopsis thaliana*	Ath‐miR167c	CaMV 35S promoter	*Arabidopsis thaliana*	Inhibited somatic embryo formation and resulted in low levels of auxin response factors 6 and 8 transcripts	Su *et al*. (2016)
*Arabidopsis thaliana*	Ath‐miR169a	CaMV 35S promoter	*Arabidopsis thaliana*	Increased leaf water loss and enhanced sensitive to drought stress	Li *et al*. (2008)
*Solanum lycopersicum*	Sly‐miR169c	CaMV 35S promoter	*Solanum lycopersicum*	Reduced stomatal conductance and transpiration rate	Zhang *et al*. (2011b)
*Solanum lycopersicum*	Sly‐miR156	CaMV 35S promoter	*Solanum lycopersicum*	Plants with reduced height, smaller but more numerous leaves and smaller fruit	Zhang *et al*. (2011b)
*Arabidopsis thaliana*	Ath‐miR169d	CaMV 35S promoter	*Arabidopsis thaliana*	Early flowering phenotype	Xu *et al*. (2013)
*Arabidopsis thaliana*	Ath‐miR169a and Ath‐miR827a	MIR gene deletion using CRISPR/Cas9 HDR	*Arabidopsis thaliana*	Efficiencies of 20% and 24% on Ath‐miR169a and Ath‐miR827a loci, respectively.	Zhao *et al*. (2016)
*Medicago truncatula*	Mtr‐miR171 h	CaMV 35S promoter	*Medicago truncatula*	Restricts root symbiosis with *Sinorhizobium meliloti*	Hofferek *et al*. (2014)
*Oryza sativa*	Osa‐miR171b and its target mimicry (STTM171b)	Ubiquitin 1 promoter	*Oryza sativa*	Thicker tillers and longer panicles with more spikelets, and plants less susceptible to *Rice stripe virus* and virus symptoms were attenuated	Tong *et al*. (2017)
*Glycine max*	Gma‐miR172a	CaMV 35S promoter	*Arabidopsis thaliana*	Early flowering phenotype	Wang *et al*. (2016)
*Glycine max*	Gma‐miR172a	CaMV 35S promoter	*Glycine max*	Tolerance to salinity	Pan *et al*. (2017)
*Glycine max*	Gma‐miR172c	CaMV 35S promoter	*Arabidopsis thaliana*	Improves drought and salinity tolerance, but increased ABA sensitivity	Li *et al*. (2016c)
*Oryza sativa*	Osa‐miR319a	CaMV 35S promoter	*Agrostis stolonifera*	Several pleiotropic phenotypes and improves drought and salinity tolerance	Zhou *et al*. (2013); Zhou and Luo (2014)
*Oryza sativa*	Osa‐miR319a and Osa‐miR319b	Rice Actin 1 and maize Ubiquitin 1 promoter, respectively	*Oryza sativa*	Several pleiotropic phenotypes and improves cold tolerance	Yang *et al*. (2013)
*Oryza sativa*	Osa‐miR319b	CaMV 35S promoter	*Oryza sativa*	Tolerance to cold	Wang *et al*. (2014)
*Oryza sativa*	Os‐miR390	CaMV 35S promoter	*Oryza sativa*	Susceptibility to cadmium	Ding *et al*. (2016)
*Oryza sativa*	Os‐miR393	CaMV 35S promoter	*Oryza sativa*	Several pleiotropic phenotypes and reduce salinity and drought tolerance	Xia *et al*. (2012)
*Arabidopsis thaliana*	Ath‐miR393	Overexpression of a miR393‐resistant TIR1 gene	*Arabidopsis thaliana*	Enhanced auxin sensitivity led to pleiotropic effects (abnormal roots development and delayed flowering) and displayed enhanced osmotic stress tolerance	Chen *et al*. (2015b, 2011))
*Arabidopsis thaliana*	Ath‐miR393a	CaMV 35S promoter	*Arabidopsis thaliana*	Tolerance for *Pseudomonas syringae*	Navarro *et al*. (2006)
*Oryza sativa*	Osa‐miR393a		*Agrostis stolonifera*	Altered phenotype (fewer amount but longer tillers, and reduced stomata density and denser cuticles) and improved tolerance to multiple stresses (drought, heat and salinity, and increased uptake of potassium)	Zhao *et al*. (2019)
*Glycine max*	Gma‐miR394a	CaMV 35S promoter	*Arabidopsis thaliana*	Drought stress tolerance	Ni *et al*. (2012)
*Arabidopsis thaliana*	Ath‐miR394a/b	CaMV 35S promoter	*Arabidopsis thaliana*	Regulation of leaf morphology, ABA hypersensitivity and ABA‐associated phenotypes and improves of drought stress tolerance	Song *et al*. (2013, 2012)
*Arabidopsis thaliana*	Ath‐miR394a	CaMV 35S promoter	*Arabidopsis thaliana*	Improved tolerance to cold stress	Song *et al*. (2016)
*Brassica napus*	Bn‐miR394	CaMV 35S promoter	*Brassica napus*	Several pleiotropic phenotypes	Song *et al*. (2015)
*Arabidopsis thaliana*	Ath‐miR395d	CaMV 35S promoter	*Brassica napus*	Alters leaf morphology and delay the transition between juvenile to adult stage of vegetative development	Huang *et al*. (2010)
*Solanum peruvianum*	Sp‐miR396a‐5p	CaMV 35S promoter	*Nicotiana tabacum*	Salinity, drought and cold stress tolerance, but increases susceptibility to *Phytophthora nicotianae*	Chen *et al*. (2015a)
*Oryza sativa*	Osa‐miR396c	CaMV 35S promoter	*Arabidopsis thaliana* and *Oryza sativa*	Less tolerance to salinity and alkali stress	Gao *et al*. (2010)
*Arabidopsis thaliana*	Ath‐miR396a and Ath‐miR396b	CaMV 35S promoter	*Arabidopsis thaliana*	Reduced susceptibility to *Heterodera schachtii*	Hewezi *et al*. (2012)
*Poncirus trifoliata*	Ptr‐miR396b	CaMV 35S promoter	*Citrus limon*	Tolerance to cold	Zhang *et al*. (2016a)
*Oryza sativa*	Osa‐miR396f	CaMV 35S promoter	Susceptible *Oryza sativa* variety	Resistance to *Dickeya zeae* (formerly known as *Erwinia chrysanthemi* pv. *zeae*)	Li *et al*. (2019)
*Arabidopsis thaliana*	Ath‐miR397	CaMV 35S promoter	*Arabidopsis thaliana*	Tolerance to chilling and freezing stresses	Dong and Pei (2014)
*Oryza sativa*	Overexpressing of Os‐miR397 contrasting with pUbi1:OsLAC3	Maize Ubiquitin 1 promoter	*Oryza sativa*	miR397 negatively regulates laccases accumulation, resulting in morphological abnormalities (feeble plants, reduced stem thickness and tendency to lodging) associated with reduced lignification	Swetha *et al*. (2018)
*Arabidopsis thaliana*	Ath‐miR398‐resistant forms of CSD1, CSD2 and CCS genes	Native promoters	*Arabidopsis thaliana*	Enhanced heat tolerance	Guan *et al*. (2013)
*Oryza sativa*	Os‐miR398	Ubiquitin 1 promoter	*Oryza sativa*	More sensitive to environmental stress	Lu *et al*. (2010)
*Arabidopsis thaliana*	Ath‐miR399d	Rd29A abiotic stress‐inducible promoter	*Solanum lycopersicum*	Tolerance to cold and phosphorus deficiency	Gao *et al*. (2015)
*Arabidopsis thaliana*	Ath‐miR399f	CaMV 35S promoter	*Arabidopsis thaliana*	Tolerance to salt stress and exogenous ABA, but hypersensitivity to drought stress	Baek *et al*. (2016)
*Arabidopsis thaliana*	Ath‐miR408	CaMV 35S promoter	*Cicer arietinum*	Tolerance to drought stress	Hajyzadeh *et al*. (2015)
*Triticum aestivum*	Ta‐miR444a	CaMV 35S promoter	*Nicotiana tabacum*	Improves the plant development	Gao *et al*. (2016)
*Populus suaveolens*	*Psu*‐miR475b	Psu‐miR475b native promoter (tissue‐specific and regulated by abiotic stress) or CaMV 35S	*Populus suaveolens*	Improved tolerance to cold stress	Niu *et al*. (2016)
*Arabidopsis thaliana*	Ath‐miR778	CaMV 35S promoter	*Arabidopsis thaliana*	Improved the roots development	Wang *et al*. (2015)
*Arabidopsis thaliana*	Ath‐miR844	CaMV 35S promoter	*Arabidopsis thaliana*	Susceptibility to *Pseudomonas syringae* and *Botrytis cinerea*	Lee *et al*. (2015)
*Arabidopsis thaliana*	Ath‐miR858a	CaMV 35S promoter	*Arabidopsis thaliana*	Improves the plant growth and early flowering	Sharma *et al*. (2016)
*Oryza sativa*	Os‐miR396c and OsGRF4 engineered resistant to Os‐miR396c	Double CaMV 35S promoter	*Oryza sativa*	Plants overexpressing OsGRF4 resistant to Os‐miR396c resulting in larger grain size and enhanced grain yield	Li *et al*. (2016b)
*Oryza sativa*	Os‐miR397a and Os‐miR397b	CaMV 35S promoter	*Oryza sativa*	Enlarges grain size and promotes panicle branching. In addition, Os‐miR397 negatively regulates OsLAC (Laccase) and results in increase of resistance to fenoxaprop‐P‐ethyl (herbicide)	Zhang *et al*. (2013); Pan *et al*. (2017)
*Arabidopsis thaliana*	Ath‐miR396	CaMV 35S promoter	*Nicotiana tabacum*	Enhances drought tolerance	Yang and Yu (2009)
*Arabidopsis thaliana*	Target mimicry of Ath‐miR858a (STTM858a)	CaMV 35S promoter	*Arabidopsis thaliana*	Reduction of plant growth and delayed flowering	Sharma *et al*. (2016)
*Arabidopsis thaliana*	Target mimicry of Ath‐miR156 (STTM156) and Ath‐miR319 (STTM319)	CaMV 35S promoter	*Arabidopsis thaliana*	Plants with smaller size and number of leaves	Franco‐Zorrilla *et al*. (2007)
–	Artificial amiR‐24	CaMV 35S promoter	*Nicotiana tabacum*	Efficient amiR‐24 targeting chitinase gene from *Helicoverpa armigera*, improving plant tolerance to caterpillar	Agrawal *et al*. (2015)
*Triticum aestivum*	Ta‐miR156, Ta‐miR166 and artificial miRNA against PDS gene	miRNA overexpression and silencing systems using a viral vector (*Barley stripe mosaic virus*)	*Triticum aestivum*	Efficient down‐regulation of Ta‐miR156 and Ta‐miR166 and overexpression of miR156 or artificial miRNA (amiRNA) targeting *phytoene desaturase* gene (amiR‐PDS)	Jian *et al*. (2017)
*Oryza sativa*	Os‐miR166 knock‐down (STTM166)	Knock‐down of miR166, using the Short Tandem Target Mimic (STTM)	*Oryza sativa*	Tolerance to drought, but causing leaf rolling and altering stem xylem development	Zhang *et al*. (2018b)
*Oryza sativa*	Os‐miR408, Os‐miR528, Os‐miR815a/b/c, and Os‐miR820a/b/c	CRISPR/Cas9 system using nonhomologous end joining (NHEJ) strategy to miRNA knockout	*Oryza sativa*	Mono and biallelic mutations generated by CRISPR/Cas9 NHEJ resulted in loss of function of target miRNAs	Zhou *et al*. (2017)
*Arabidopsis thaliana*	Ath‐miR408	Overexpressing using CaMV 35S or BFN1 promoters	*Arabidopsis thaliana*	Enhances tolerance to cold, oxidative and salinity stresses, but reduces the tolerance to osmotic and drought stresses	Ma *et al*. (2015)
*Oryza sativa*	Os‐miR528	CaMV 35S promoter	*Agrostis stolonifera*	Plants displayed shortened internodes, increased tiller number, upright growth, tolerance to salinity stress and nitrogen starvation	Yuan *et al*. (2015)
*Arabidopsis thaliana*	Ath‐miR159b	CRISPR/Cpf1 (deactivated) fused to three SRDX repressors to targeting the promoter of miR159b and cause down‐regulation of the miR159b	*Arabidopsis thaliana*	Lower expression of miR159b in T1 transgenic lines	Tang *et al*. (2017)
*Solanum pimpinellifolium*	Sp‐miR482b and its target mimicry (STTM482)	CaMV 35S promoter	*Solanum lycopersicum*	Sp‐miR482b overexpression resulted in more severe disease symptoms, while silencing enhanced tomato resistance to *Phytophthora infestans*	Jiang *et al*. (2018)
*Solanum tuberosum*	St‐miR482e	CaMV 35S promoter	*Solanum tuberosum*	Susceptibility to *Verticillium dahliae*	Yang *et al*. (2015)
*Oryza sativa*	Os‐miR529a	Maize Ubiquitin 1 promoter	*Oryza sativa*	Resistance to oxidative stress, resulting in increased seed germination rate, root tip cell viability, reduced leaf rolling rate and chlorophyll retention	Yue *et al*. (2017)
*Hordeum vulgare*	Artificial and engineered Hvu‐miR171 targeting viral genes	CaMV 35S and maize polyubiquitin promoters	*Nicotiana benthamiana* and *Hordeum vulgare*	Resistance to *Wheat dwarf virus*	Kis *et al*. (2016)
*Oryza sativa*	Rice lines overexpressing target mimicry to modulation of multiples miRNA and miR398 overexpressing	Maize Ubi 1 promoter	*Oryza sativa*	Os‐miR398 modulation increases panicle length, grain number and size. In addition were discovered additional agronomic functions for several known miRNAs (e.g. Os‐miR172 and Os‐miR156)	Zhang *et al*. (2018a)
*Glycine max*	Overexpression of Gma‐miR482, Gma‐miR1512 and Gma‐miR1515	CsVMV promoter for constitutive expression and soya bean ENOD40 promoter for nodulation‐inducible expression	*Glycine max*	Significant increases of nodule numbers, while root length, lateral root density and the number of nodule primordia were not altered	Li *et al*. (2010)
*Arabidopsis thaliana* and *Hordeum vulgare*	Ath‐miR827 and Hvu‐miR827	CaMV 35S and Zm‐Rab17 promoters, respectively	*Hordeum vulgare*	CaMV 35S:Ath‐miR827 resulted in pleiotropic effects in both well‐watered or drought‐treated plants, while Zm‐Rab17:Hvu‐miR827 resulted in tolerance to drought	Ferdous *et al*. (2017)
*Solanum lycopersicum*	Target mimicry of Sly‐miR482b, c and e (STTM482) and Sly‐miR2118a and b (STTM2118)	Overexpressing of STTM482 and STTM2118 driven by double 35S promoter	*Solanum lycopersicum*	Enhanced resistance to bacterial and oomycete pathogens	Canto‐Pastor *et al*. (2018)

The overexpression, up‐ or down‐regulation or knock‐in of transcribed MIR gene sequences has confirmed the involvement of miRNAs in stress responses in different plant species. For example, MIR gene overexpression with constitutive promoters (e.g. *Cauliflower mosaic virus* (CaMV) 35S, maize ubiquitin 1 and rice actin 1) has produced desirable agronomic traits, such as drought, cold, heat and salinity tolerance and resistance to pathogens (Table 1). Additional examples of patented inventions using miRNA overexpression include improved resistance to cyst nematodes (miR164 and miR396; WO2012058266 A1 and WO2012149316 A2), tolerance to drought (miR166; CN102250903A) and salinity (miR397; WO2007103767 A2), artificial MIR genes (US8536405 B2, WO2009079548 A3) and target mimicry (EP2873735 A1, WO2012056401A1).

However, strong constitutive overexpression often causes undesirable pleiotropic effects because miRNAs are frequently involved in the regulation of a number of miRNAs (Ferdous *et al*., 2017). The overaccumulation of certain miRNAs alters the expression of essential target genes involved in plant development, which may produce undesirable phenotypes (Trumbo *et al*., 2015). Greater target specificity can be achieved with overexpression driven by tissue‐specific (Niu *et al*., 2016) or stress‐inducible (Gao *et al*., 2015) promoters. Additional strategies include the overexpression of target mRNAs that are resistant to specific miRNAs (Guan *et al*., 2013), the expression of artificial target mimics that can cancel out the effect of endogenous miRNA activity (Sharma *et al*., 2016) and the expression of artificial MIR gene targeting only the desired mRNAs (Agrawal *et al*., 2015). Jian *et al*. (2017) reported a new method for miRNA overexpression or knock‐down based on a viral vector (*Barley stripe mosaic virus*) that can be used as a probe to investigate the activities of miRNAs (Table 1). More recently, genome‐editing technologies based on the CRISPR/Cas9 or CRISPR/Cpf1 system have revealed new insights into miRNA fine‐tuning, which has been employed in the editing (Zhou *et al*., 2017) or transcriptional regulation (Lowder *et al*., 2015; Tang *et al*., 2017) of MIR genes. Next, we present and discuss the advantages and limitations of each of these strategies.

## Biotechnological approaches to fine‐tuning of miRNA activity

### Transgenesis, cisgenesis and intragenesis

The transference of one or more MIR genes between noncrossable plant species (transgenesis) or between crossable or the same species (cisgenesis and intragenesis) has been successfully performed in several plant species (Table 1). In addition, conceptually, any element used in T‐DNA can be engineered to enhance transgene expression, or the same native sequence can be used to maintain the original cisgene features. Unlike cisgenesis, intragenesis employs hybrid sequences (optimized genes and/or additional promoter and terminator sequences) from sexually compatible species (Holme *et al*., 2013). For example, strong constitutive (mostly CaMV 35S), strong endogenous (Lu *et al*., 2010; Yang *et al*., 2013) or native tissue‐specific or stress‐induced (Niu *et al*., 2016) promoters were exploited to overexpress MIR genes (Table 1). However, the manipulation of MIR genes using any of these strategies, especially overexpression driven by a strong promoter, has also resulted in undesirable traits, such as pleiotropic phenotypes (Table 1). This result can be explained by the involvement of miRNAs in diverse and complex regulatory networks. Strong overexpression impairs the fine‐tuning of several biological pathways, which might be overcome using specific promoters (e.g. tissue‐specific, stress‐induced or developmental stage‐specific promoters).

### Artificial MIR genes

**Figure 2:**
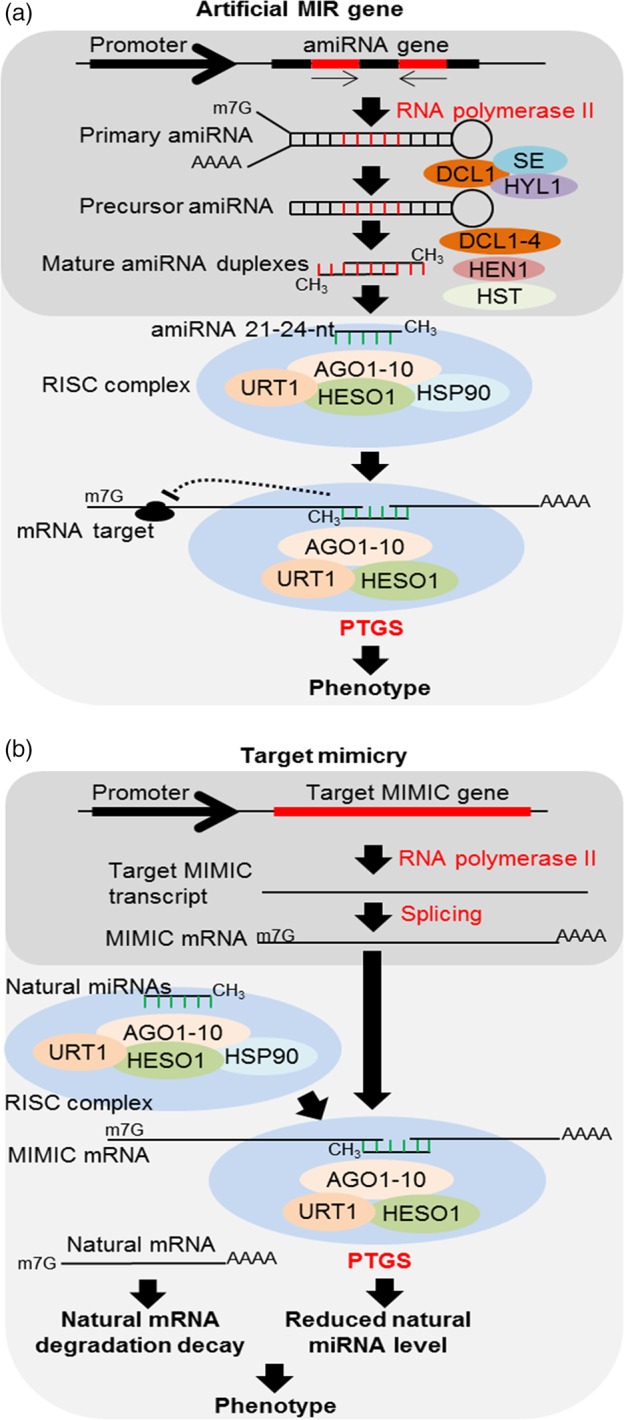
Constitutive or transient expression of (a) artificial MIR genes to accumulate artificial miRNA (amiRNA) and the depletion of the target mRNA (Zhang *et al*., 2018a). In brief, the amiRNA gene under the control of a typical promoter is transcribed by RNA polymerase II in the nucleus, and primary transcripts are similarly processed to canonical miRNAs, originating primary amiRNA (pri‐amiRNA). The pri‐amiRNA is processed by DCL1, SE and HYL1, originating precursor amiRNA (pre‐amiRNA), which are processed again preferentially by DCL1, resulting in the increased accumulation of 21‐nt amiRNA duplexes. However, it is not yet clear whether DCL2‐4 also acts on this pre‐amiRNA. Niu *et al*. (2006) showed that DCL1 development has a major effect on pre‐amiRNA. However, DCL1 knockout plants (*dcl1*) also showed the accumulation of these 21‐nt amiRNAs, although comparatively low accumulation was observed. This finding may suggest that DCL2‐4 can also act in pre‐amiRNA processing. Then, these 21‐nt amiRNAs are methylated by HEN1, which subsequently undergoes nucleus–cytoplasm transport by HST to P‐bodies and becomes integrated into the RISC, where it will act in a manner similar to canonical miRNAs on PTGS. (b) Target mimicry strategy to deplete specific miRNAs (Peng *et al*., 2018; Zhang *et al*., 2017). The constitutive or transient expression of the target mimic gene driven by a specific promoter is transcribed by RNA polymerase II in the nucleus. The primary transcripts are processed with the addition at 5′ terminus of a 7‐methyl guanosine cap, 3′ polyadenylation and RNA splicing, originating primary mimic mRNA, which is then transported to the cytoplasm, thereby depleting the miRNAs.

Currently, various strategies exploit PTGS to down‐regulate or uncover the functions of specific genes (Figure 2a). These approaches are primarily based on the accumulation of siRNAs derived from dsRNAs using a virus‐induced gene silencing model (VIGS) and by the constitutive overexpression of an engineered target gene (full or partial length) in the antisense or sense/antisense orientation. However, these approaches produce a diverse set of siRNAs that might potentially silence nontarget genes (resulting in off‐target effects). To overcome this problem, an artificial MIR gene (amiRNA) strategy was developed to produce specific miRNAs and effectively silence target genes (Zhang *et al*., 2018a). These amiRNAs have a conserved secondary foldback structure similar to that of a typical pre‐miRNA. Nonetheless, the original miRNA‐5p:miRNA‐3p sequence is replaced by an engineered miRNA targeting a specific mRNA. Thus, amiRNAs can be engineered to target any mRNA with higher specificity compared to strategies based on dsRNA overexpression or siRNA accumulation. Pre‐amiRNA processing typically results in a single amiRNA targeting for a known sequence, thus helping to avoid off‐target effects. In addition, the systemic movement of these mature amiRNAs is restricted in some cases (Carlsbecker *et al*., 2010), and the production of secondary siRNAs from pre‐amiRNA sequences is very limited (Manavella *et al*., 2012). Furthermore, amiRNAs are stable and inheritable. The main drawbacks of this strategy are the selection of backbone or pre‐amiRNA sequences for effective silencing without any off‐target effects (Carbonell *et al*., 2015). For major target specificity, mature amiRNAs must have low sequence similarity with nontarget genes (Zhang *et al*., 2018a).

In addition to the control of endogenous gene expression and the study of the role of new MIR genes, an amiRNA strategy has also successfully been used to knock out genes from insect pests, nematodes, viruses and other phytopathogens (Kis *et al*., 2016; Wagaba *et al*., 2016). Similarly, Arabidopsis expressing two amiRNAs targeting the mRNA of the P69 and HC‐Pro proteins showed resistance to *Turnip yellow mosaic virus* and *Turnip mosaic virus*, respectively (Niu *et al*., 2006). Agrawal *et al*. (2015) showed that tobacco overexpressing an engineered amiRNA was resistant to *Helicoverpa armigera*.

Ju *et al*. (2017) developed a new overexpression system for artificial and endogenous miRNAs and siRNAs based on delivery from a viral satellite DNA vector for functional analysis in plants. This viral system generated promising results for the overexpression of endogenous or artificial MIR gene, siRNAs and trans‐acting siRNAs in *Nicotiana benthamiana*.

### Endogenous and artificial target mimicry

Similar to the overexpression of MIR genes, the negative regulation of the accumulation of some specific miRNAs allows the achievement of desirable agronomic characteristics. Endogenous target mimicry (eTMs) is another natural mechanism involved in modulating miRNA accumulation, controlling several biological processes in plants (Karakülah *et al*., 2016; Lin *et al*., 2015). These eTMs are innumerous long noncoding RNA (lncRNA) or circular noncoding RNAs (circRNAs) transcribed from genome and differential expressed usually in response to stresses or other adverse conditions (Karakülah *et al*., 2016; Liu *et al*., 2017). It acts as natural SPONGES mainly for quick fine‐tune of miRNAs in plant response or adaptation to a new condition. To reproduce this mechanism, an artificial short tandem target mimic (STTM) strategy was developed to modulate miRNAs accumulation and improve desirable agronomic traits. This strategy is based on the transient or constitutive overexpression (driven by constitutive, tissue‐specific or induced promoters) of an engineered lncRNA with high nucleotide sequence identity to target mRNA (Reichel and Millar, 2015; Figure 2b). Similarly, the overexpression of synthetic circRNA is also suggested as potential alternative for miRNA modulation in plants. These STTMs contain two or more conserved binding site for the specific target miRNA, but with three nucleotide mismatches at the miRNA‐cleavage site, which prevent its cleavage, whereas the miRNA remains hybridized and biologically inactivated. Thus, STTM sequester miRNAs from the endogenous target mRNA resulting in its up‐regulation (Franco‐Zorrilla *et al*., 2007). Several STTMs targeting the MIR genes in model and crop plants have been recently engineered and constitutively expressed as transgenes for the comprehensive functional analysis of miRNAs (Peng *et al*., 2018; Zhang *et al*., 2017). In addition, STTMs have been optimized to enhance loss‐of‐function phenotypes caused by artificial single target mimics. Similarly, miRNA SPONGES are another synthetic transcript produced from transgenes with activity similar to STTMs. These RNAs SPONGES contain multiple miRNA binding sites *in tandem* repeated and are often used to inhibit whole families of related miRNAs in both plant and animal systems (Reichel *et al*., 2015; Thomson and Dinger, 2016). For example, Jiang *et al*. (2018) improved tomato resistance to *Phytophthora infestans* using a STTM strategy to silence miR482b from *Solanum lycopersicum*.

### MIR gene editing using endonucleases

Meganucleases and zinc‐finger proteins (ZNFs) were the first endonucleases engineered for plant genome editing. Meganucleases recognize double‐strand DNA sequences of 12–42 nucleotides in length in a highly specific manner, consequently restricting the number of targets. In contrast, ZNFs consist of two modules of tandem repeat DNA‐binding domains flanking the *Fok*I nuclease catalytic domain (Osakabe and Osakabe, 2015). Each of these domains recognizes a unique nucleotide triplet, while each module has specificity for ≥24 nucleotides. Similar to ZNFs, transcription activator‐like effector nucleases (TALENs) comprise two modules of tandem repeat DNA‐binding motifs flanking a *Fok*I motif. However, each TALEN DNA‐binding motif recognizes a single nucleotide. Recently, clustered regularly interspaced short palindromic repeats/CRISPR‐associated protein‐9 nuclease (CRISPR/Cas9), CRISPR/Cpf1 or CRISPR/Csm1 systems, a new nuclease class guided by RNA (guide RNA), have been optimized for plant genome editing (Osakabe *et al*., 2016; Granted patent US9896696B2; Wang *et al*., 2018a). Both nucleases cause double‐strand breaks (DSBs) at the target site, and during DSB repair, the insertion or deletion (*indels*) of nucleotides may occur. In addition to *indels*, nucleotide‐specific editing can be achieved using engineered donor DNA.

**Figure 3:**
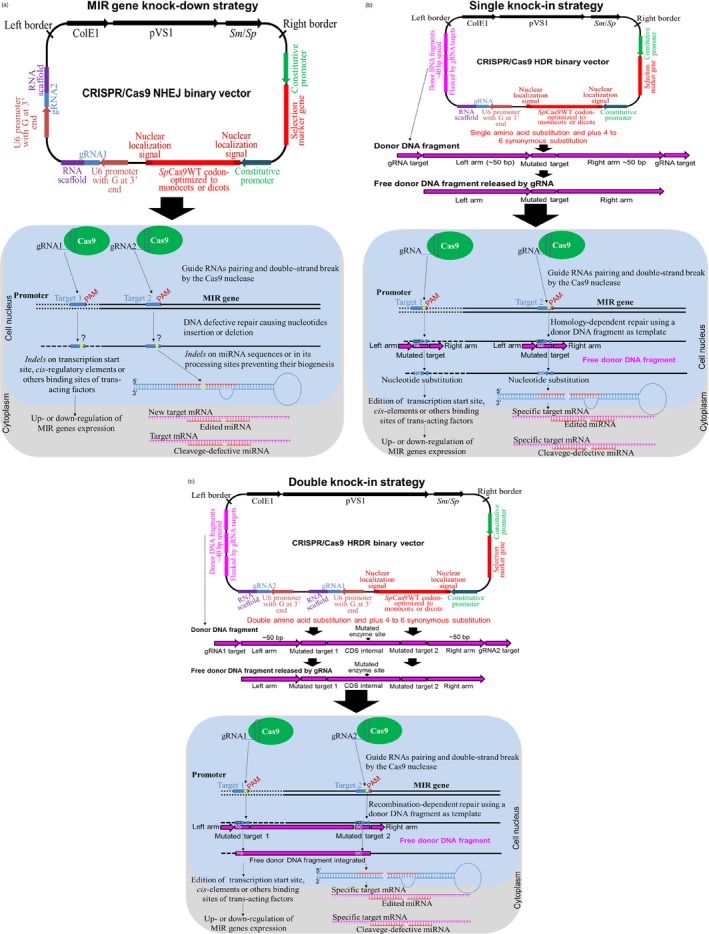
Genome editing using CRISPR/Cas9 system. (a) The CRISPR/Cas9 system to target promoter sequences or generate MIR gene knock‐down using nonhomologous end joining (NHEJ) strategy. Above, a typical CRISPR/Cas9 NHEJ binary vector carrying a T‐DNA that contains a selection marker gene under control of a constitutive promoter, *Streptococcus pyogenes* Cas9 wild‐type gene (*Sp*Cas9WT) codon‐optimized to monocots or dicots and containing one or two nuclear localization signals under control of a constitutive promoter, one or two RNA guide RNAs (gRNA1 or gRNA2) and an RNA scaffold *in tandem* under control of the U6 RNA polymerase III promoter containing a guanine (G) extra at the 3′ end. Below, an overview of the NHEJ strategy in plants containing T‐DNA from the CRISPR/Cas9 binary vector integrated into the genome (constitutive expression) or by transient expression (e.g. in protoplasts) via biolistic approach or the type III secretion system of *Agrobacterium tumefaciens* (Chang *et al*., 2016; Zhou *et al*., 2017). Below, the complex gRNA:RNA scaffold is transcribed, associated with Cas9 nuclease in the nucleus and directed to the target sequence in genomic DNA. The gRNAs match the target sequence (promoter sequence or MIR gene) and mediate its cleavage by Cas9 nuclease next to the protospacer adjacent motif (PAM), generating a double‐strand DNA break (DSB). After DNA cleavage, the damage is corrected by the DNA repair mechanism of the plant cell, but errors (insertion or deletions of any nucleotides, named *indels*) can be inserted in the repaired DSB sequence, resulting in *indels* within the transcription start site, cis‐regulatory elements or other binding sites of trans‐acting factors, leading to the up‐ or down‐regulation of MIR gene expression. In addition, *indels* in miRNA processing sites prevent the biogenesis of these molecules. (b) CRISPR/Cas9 system to target promoter sequences or generate a MIR gene single knock‐in using a homology‐directed repair (HDR) strategy. Above, a typical CRISPR/Cas9 HDR binary vector carrying a T‐DNA, similar to NHEJ, but containing only one gRNA and one to three copies of the donor DNA fragment engineered to match the target DNA sequence. These donor DNA fragments are flanked by the target sequences of the gRNAs at the 5′ and 3′ terminus, homology arms that flank the target site and mutated target sequence containing amino acid substitutions plus 4–6 synonymous substitutions, which prevent the edited DNA from being paired by the gRNA and cleaved by Cas9 nuclease. These donor DNA molecules can be delivered by transgene integration into the genome and released by the gRNA or by a biolistic approach using free donor DNA (donor DNA fragment without gRNA target), which will serve as a repair template for damaged DNA. Below, the complex gRNA:RNA scaffold is transcribed, associated with Cas9 nuclease in the nucleus and directed to the target gene sequence in the genome. The gRNAs match the target sequence (promoter sequence or MIR gene) and mediate its cleavage by the Cas9 nuclease next to the PAM sequence, generating DSB. After DNA cleavage, this damage is corrected by the DNA repair mechanism using the free donor DNA fragment as the repair template based on the homology sequence, resulting in nucleotide exchange and amino acid substitution (Sun *et al*., 2016; Zhao *et al*., 2016). (c) CRISPR/Cas9 system to target promoter sequences or generate simultaneous MIR gene double knock‐in using a homologous recombination‐directed repair (HRDR) strategy. Above, a typical CRISPR/Cas9 HRDR binary vector carrying a T‐DNA, similar to NHEJ and HDR, containing two gRNAs and donor DNA fragments engineered to simultaneously edit two interspaced target sites (Sun *et al*., 2016; Zhao *et al*., 2016). In brief, DSB is repaired by the DNA repair mechanism using the free donor DNA fragment as the repair template. In this case, the donor DNA fragment is integrated into the target site by homologous recombination.

CRISPR/Cas9 nonhomologous end joining (NHEJ; Figure 3a) can be achieved by the introduction of *indels* at pre‐miRNA sequences or the miRNA processing sites of MIR genes, which impedes or retards miRNA biogenesis (Chang *et al*., 2016; Zhou *et al*., 2017). Similarly, *indel* insertion in target genes can interfere with miRNA‐target mRNA pairing and lead to the subsequent failure of mRNA cleavage into RISC. In addition, homology‐directed repair (HDR) (Figure 3b) and homology and recombination‐directed repair (HRDR; Figure 3c) can be achieved by the full deletion or knock‐in of MIR genes or their promoter sequences (Zhao *et al*., 2016).

However, the knock‐down or knock‐in of MIR genes is challenging compared to the modification of protein‐encoding genes due to the complexity of the regulatory networks, the reduced length for gRNA design or targeting, and the fact that miRNA is encoded within intron sequences, which hinders MIR gene editing (Barrangou *et al*., 2015; Basak and Nithin, 2015). Additionally, the length of MIR genes reduces the number of possible gRNA/Cas9 targets, thus reducing the odds of finding a target near the mature miRNA (Jacobs *et al*., 2015). In contrast, CRISPR/Cas9‐mediated knock‐down can be more efficient considering homologous, orthologous or paralogous MIR genes and pri‐miRNA regions (Barrangou *et al*., 2015).

**Figure 4:**
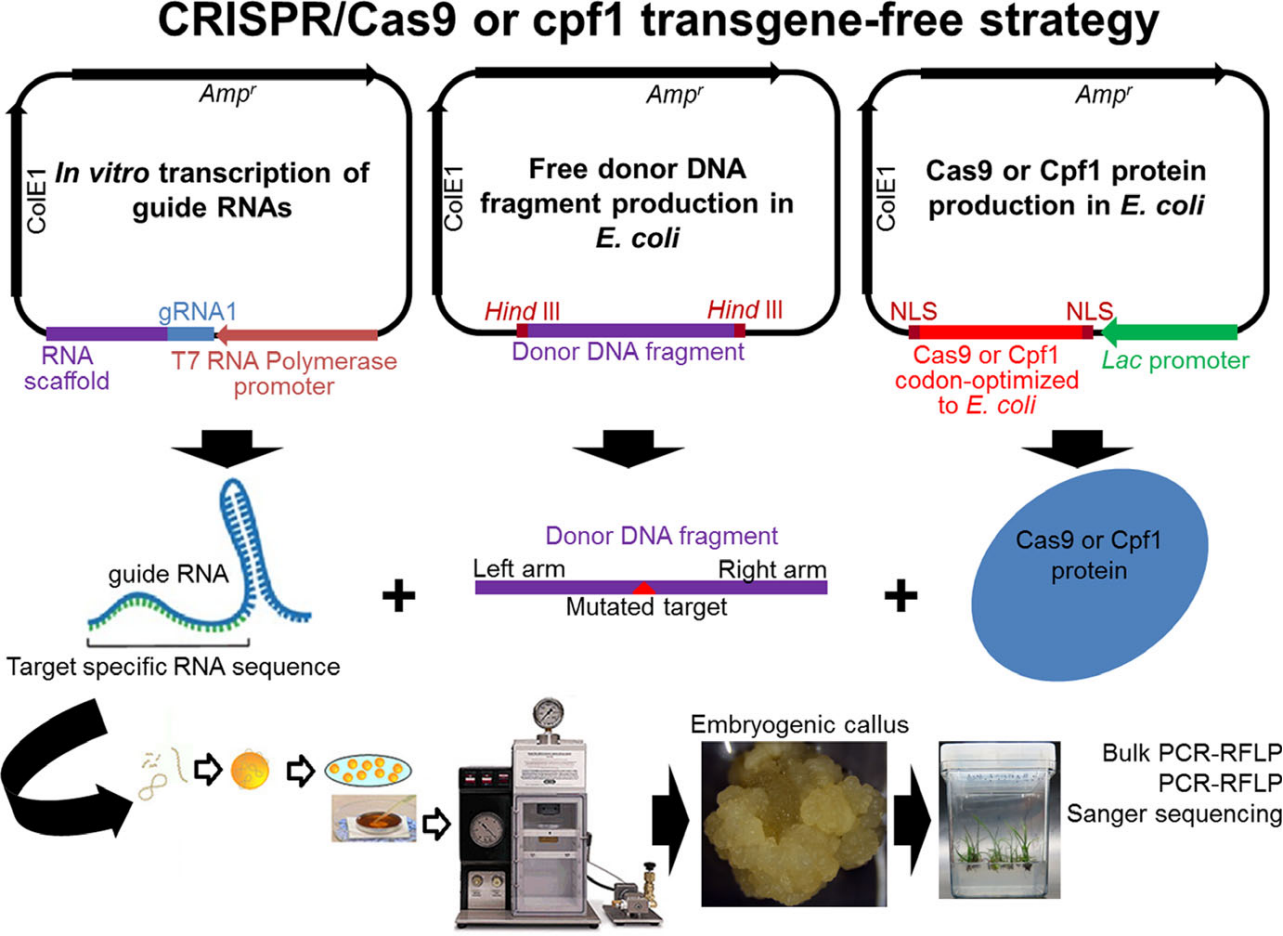
CRISPR/Cas9 or CRISPR/Cpf1 system to target genes or generate MIR gene knock‐down or knock‐in using similar NHEJ, HDR or HRDR strategies, but transgene‐free. Above, CRISPR/Cas9 vectors for *in vitro* (e.g. *Escherichia coli*) production of guide RNAs (gRNA), free donor DNA fragment and Cas9 or Cpf1 protein flanked by one or two nuclear localization signals (NLS) (Liang *et al*., 2017). After purification, the gRNA, nuclease protein and donor DNA fragment are mixed and assembled in microcarrier particles (tungsten or gold) and delivered into plant cells (e.g. embryogenic callus, apical meristem or protoplasts) using a biolistic particle delivery system.

Typical binary vectors for the CRISPR/Cas9 system using a transgenic approach basically contain one selection marker, a Cas9 nuclease sequence that is codon‐optimized to monocots or dicots and flanked by two nuclear localization signals, and gRNAs under the control of a specific promoter (Figure 3a–c). These vectors can also contain donor DNA fragments in tandem repeats, which are used as repair moulds of DSB in HDR and HRDR strategies (Figure 3b, c). For the CRISPR/Cas9 or Cpf1 system using a transgene‐free strategy (without transgene integration into the plant genome), the nuclease and gRNA are synthetized *in vitro* and then biolistically delivered into plant cells (Liang *et al*., 2017; Figure 4).

Jacobs *et al*. (2015) showed that CRISPR/Cas9 successfully targeted soya bean miR1514 and miR1509 using biolistic delivery for the transient expression of the CRISPR/Cas9 vector harbouring Cas9 nuclease and gRNAs. Similarly, Li *et al*. (2016a) induced specific mutations in the miR156 recognition site of the rice *ipa1* gene (*ideal plant architecture 1*), which improved several traits related to plant architecture. Zhou *et al*. (2017) successfully generated mono‐ and biallelic mutations in several MIR genes of T0 rice lines, resulting in loss of function for target miRNAs.

**Figure 5:**
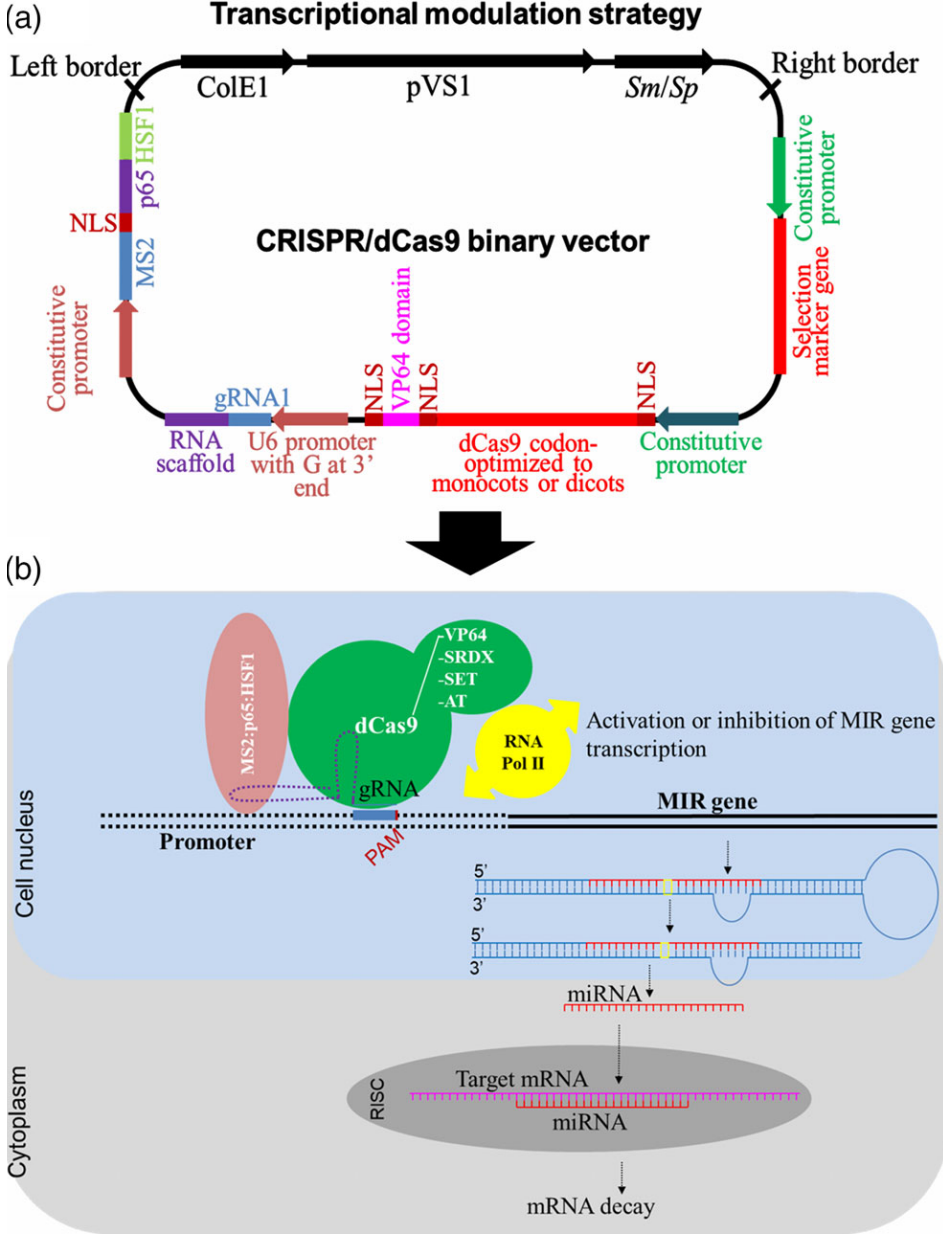
Transcriptional modulation (activation or repression) of MIR genes using CRISPR/dCas9 system. Above, a typical CRISPR/dCas9 binary vector carrying a T‐DNA that contains a selection marker gene driven by a constitutive promoter; dead Cas9 (dCas9) gene codon‐optimized to monocots or dicots and containing one or two nuclear localization signals (NLS) fused *in tandem* with dCas9:VP64, dCas9:SDRX, dCas9:SET or dCas9:AT functional domains, and also driven by a constitutive promoter; one RNA guide (gRNA) and an RNA scaffold *in tandem* driven by U6 RNA polymerase III promoter containing a guanine (G) extra at the 3′ end; and MS2, p65 and HSF1 activation domains fused *in tandem* and containing one internal NLS driven by a constitutive promoter (Lowder *et al*., 2018; Tang *et al*., 2017). Below, an overview of the dCas9:VP64, dCas9:SDRX, dCas9:SET or dCas9:AT coupled with guide RNA and MS2:p65:HSF1 is directed to promoter region of MIR gene controlling the MIR gene transcription.

In addition to MIR genes, the CRISPR/Cas9 system can be used for editing promoter or enhancer sequences (Chang *et al*., 2016; Zhou *et al*., 2017). A challenge yet to be overcome is the disruption or alteration of transcription start sites, cis‐regulatory elements (e.g. TATA‐ or GC‐box) or other binding sites of trans‐acting factors (e.g. ABRE and DRE motifs) to achieve precise miRNA expression that improves tolerance without pleiotropic effects. Furthermore, CRISPR/Cas9 technology has recently been used to modulate gene expression through the activation or transcriptional repression of target genes. To this end, a deactivated Cas9 nuclease (dCas9), lacking the two domains needed for DNA double‐strand cleavage (D10A/H840A), has been successfully used (Figure 5). The dCas9 is incapable of cleaving any DNA but is successfully guided to the promoter sequence of desirable MIR genes. In contrast, it can be fused to other functional domains, for example dCas9:VP64 (quadruple tandem repeat of the *Herpes simplex virus* VP16‐activation domain), dCas9:SRDX (synthetic transcriptional repressor pco‐dCas9‐3X) or dCas9:SET (methyltransferase domain of the H3K9me3 writer) and dCas9:AT (acetyltransferase domain), which act as transcriptional activators (Chavez *et al*., 2015), repressors (Lowder *et al*., 2017) or epigenetic modifiers (O'Geen *et al*., 2017), respectively. The transcriptional modulation starts by dCas9 (or dCfp1) guided by gRNA to sequences immediately upstream of the transcriptional start site (TSS) of an MIR gene. In addition, the fused MS2‐p65‐HSF1 activation domains are simultaneously overexpressed, which will interact with the stem‐loop of gRNA and recruit additional transcriptional factors to this promoter, improving its transcriptional level (Lowder *et al*., 2015). Several promising results already have been obtained using this approach, allowing further expansion of the applications of this technology (Lowder *et al*., 2018; Park *et al*., 2017). Tang *et al*. (2017) used the CRISPR/Cpf1 system to demonstrate the efficient transcriptional repression of miRNA159b using deactivated Cpf1 (without the domain of DNA cleavage) fused to the SRDX transcriptional repressor domain.

Furthermore, CRISPR/Cas13a use novel nuclease type (class II type VI‐A endoribonuclease) also guided by gRNA to targeting and cleaving single‐stranded RNA (ssRNA or mRNA). LwaCas13a from *Leptotrichia wadei* contain two nucleotide‐binding domains (2× HEPN) associated with different RNase activity. It has been successfully established in mammalian and plant cells to knock‐down of any exogenous or endogenous RNA (e.g. immunity against viral RNA, and single or multiple knock‐down) and thus enable numerous approaches with RNA biology (Abudayyeh *et al*., 2017; Aman *et al*., 2018). In contrast to usual RNAi system, CRISPR/Cas13a system also has activity in nuclear RNAs and greater target specificity. In addition, point mutation in HEPN domains abolished its nuclease activity (dead LwaCas13a or dCas13a), expanding the possibilities of its use (East‐Seletsky *et al*., 2017). However, CRISPR/Cas13a has not yet been established for pre‐, pri‐miRNA or mature miRNA editing in plants. On the other hand, LwaCas13a or dCas13a has great potential to edit its transcripts by knock‐down (e.g. miRNA binding, cleavage and degradation) or loss of function (e.g. affecting miRNA/mRNA‐binding sites, both in miRNA and in mRNA). Similarly, eTMs modulation using CRISPR/Cas13a allows to increase the accumulation of specific miRNAs. In other context, dCas13a can be fused with a deaminase domain (e.g. ADAR2 domain to adenosine‐to‐inosine deaminase, or dCMP domain to cytidine‐to‐uridine deaminase) and used to edit polymorphisms/mutations in pri‐, pre‐miRNA or miRNA sequences for various biological purposes (Cox *et al*., 2017). The LwaCas13a or dCas13a expression driven by tissue‐specific or induced promoters, or in viral vectors can allow more precise and consistent modulation of this target RNA. At least, several orthologous Cas13 nucleases are being characterized from other bacterial species, such as PspCas13b from *Prevotella* sp. which showed higher levels of RNA knock‐down compared to LwaCas13a (Cox *et al*., 2017).

### Topical delivery of pri‐miRNA or mature miRNA

RNAi technology using transgene‐free approach was recently optimized from topical delivery (foliar sprays) of the nanostructured and stabilized dsRNA molecules in model or crop plants to pathogens control or insect pest management (Joga *et al*., 2016; McLoughlin *et al*., 2018). Carrier nanoparticle (e.g. biopolymers of chitosan, silicon, carbon and clay nanosheets), ribonucleoprotein particle (e.g. peptide transduction domain–dsRNA binding domain) and cross‐linkers (e.g. tripolyphosphate, dextran sulphate and poly‐D‐glutamic acid) were successfully optimized to improve the delivery and internalization of highly integrated RNA in plant cell (Cunningham *et al*., 2018). However, use of this technology for the delivery of pri‐miRNA or mature miRNA aiming the modulation of endogenous genes to improve agronomic traits or cross‐control of insect pest or pathogens is still being established in plants. The higher stability and internalization potential of the pri‐miRNA compared to mature miRNA provides possibilities for manipulation of the transcriptional profile of adult plants without the use of transgenics. In respect to cross‐control of insect pests, delivery of structured pri‐miRNA can prevent its processing in the host plant (avoiding plant off‐target modulation and pleiotropic effects) and the acquisition by the insect in this host plant results in natural delivery. These structured pri‐miRNAs are viroid‐like engineered molecules flanked by pH‐dependent ribozymes domain, which are not processed by the RNAi machinery of plant, but are efficiently processed into insect digestive tract and cells (patent application from INPI under number: BR102017006904‐4; Maria Fatima Grossi‐de‐Sa, personal communication, 20 February 2019). Currently, the cost of large‐scale production of dsRNA is the major bottleneck; however, there are already some private companies that supply these molecules, nanoparticles and stabilizing compounds.

### Epigenetic memory

Plants are frequently exposed to different and/or concomitant stresses, and these conditions trigger defence responses that minimize the negative effects of additional stress. The defence responses of plants are enhanced by retaining “molecular memories” of previous stress events through epigenetic mechanisms (Crisp *et al*., 2016). This epigenetic memory allows subsequent defence or adaptation responses to be more efficient upon exposure to the same stress. In some cases, this epigenetic information can be transmitted from generation to generation (Crisp *et al*., 2016; Kinoshita and Seki, 2014; Liu *et al*., 2015). The siRNA or miRNA‐directed DNA methylation and histone modifications, which include methylation, phosphorylation, acetylation, ubiquitylation and sumoylation, are the main epigenetic modifications at genomic regions that contain regulatory sequences (e.g. transcription regulatory sequences), protein coding genes or MIR genes (Xie and Yu, 2015). The 24‐nt miRNAs are considered one of the primary modes for the epigenetic modulation of the genome, which in turn modulates the expression of several genes or MIR genes involved in different biological processes (Crisp *et al*., 2016). Thus, epigenetic modifications in the genome from biotic or abiotic stress events act indirectly inhibiting or activating the transcription of innumerable MIR genes and eTMs involved in the networks of responses to these stresses.

### Interkingdom cross‐talk by the horizontal transference of miRNAs

siRNA uptake from oral delivery and successful modulation of endogenous target through the process of RNAi was showed by first time in *Caenorhabditis elegans*. The horizontal transfer of miRNA until recently was filled of controversies both in the passive context (voluntary transfer during interaction, or by uptake and secretion during feeding in the cell of host organism) and active (release or ingestion of miRNAs by the host or parasite organism as a form of defence or counter‐defence). Transgenic plants overexpressing dsRNA molecules and consequently accumulating high amount of siRNAs that target insect pest or nematodes genes were successful developed and displayed improved resistance to these parasite organisms (Zotti *et al*., 2018). However, it has been observed the requirement of dsRNA delivery with longer length for successful processing by the RNAi machinery of insect pest and efficient knock‐down of its target genes (Maria Fatima Grossi‐de‐Sa, personal communication, 20 February 2019). This suggests that although RNAi machineries exhibit innumerable interkingdom similarities, they also have important specificities. In addition, it is known that any type of RNA outside its natural cellular context is easily and rapidly degraded or inactivated. The presence of numerous types of exo‐ and endonucleases prevents the occurrence of any aberrant event in the host cell, both for endogenous and exogenous RNA. On the other hand, the successful delivery of at least some dsRNA, siRNA, pri‐miRNA or mature miRNA molecules may occur; however, will they in fact act on the modulation of endogenous genes? In fact, it has been scientifically proven in the last years that the uptake of miRNAs and the regulation of target genes in host organisms are possible during compatible or incompatible interactions (Wang *et al*., 2018b; Zhang *et al*., 2012). In addition, high stability of pre‐ and pri‐miRNA suggests their greater probability of being delivered successfully in the cells of parasite or parasitized organisms, but they will in fact be properly processed by the RNAi machinery of these organisms. It is believed that in some cases is possible, since that these molecules are compatible with RNAi machinery of the recipient organism. Unfortunately, sensitive methods, appropriate approaches and efficient protocols needed for elucidation of the pri‐miRNA or mature miRNA interkingdom cross‐talk are yet very limited. The NGS technologies helped by powerful bioinformatics tools have enabled to access more easily and precisely of the uptake and secretion of miRNA during host‐parasit interaction. In addition, these approaches also allow to observe the regulation of endogenous genes by these miRNA (Dickinson *et al*., 2013; Chen *et al*., 2013). Furthermore, can these delivered miRNAs act as effector molecules modulating host defence mechanisms or in counter‐defence? Weiberg *et al*. (2013) showed that *Botritys cinerea*‐siRNAs are successful delivered in *Arabidopsis thaliana* cells during pathogenesis, binding to AGO1 and selectively silencing host immunity genes. Similarly, Zhang *et al*. (2016b) showed that in response to infection with *Verticillium dahliae*, cotton plants increase accumulation of Gh‐miR166 and Gh‐miR159, which were exported to the fungal hyphae for specific silencing of virulence genes. In this same context, can miRNA cross‐talk also play a role in epigenetic feedback? In plants, there is still no concrete evidence for this, but some studies show the presence of numerous miRNAs in milk of human or animals. In this context, Perge *et al*. (2017) speculate that milk‐derived miRNAs secreted in exosomes might be involved in the epigenetic reprogramming of the children. This would suggest that this mechanism may indeed also occur in plants. It is believed that this mechanism would be acquired evolutionarily from an interaction between individuals and that would act both in reprogramming of host defence and parasite counter‐defence.

In respect to eTMs cross‐talk, can they also act as exogenous SPONGES in parasite or parasitized organisms? On the one hand, eTMs are single‐strand RNA extremely susceptible to the extracellular environment or to ribonucleases. However, could indeed its successful delivery trigger the regulation of endogenous miRNAs? Despite possible delivery success of eTMs, they have usually single binding site for sequence‐specific miRNAs, thus restricting their action spectrum in other nontarget organisms. Unlike, circRNAs are more stability and can in fact be more active in this cross‐talk. Until then, it is known that any exogenous RNA captured by the cell is rapidly destroyed by the RNAi machinery and a secondary siRNA‐based regulatory signal is then amplified, making this receptor cell even more prepared to destroy new molecules of this invasive RNA. For example, plant viruses efficiently bypass this primary defence mechanism of host by encoding suppressor proteins of the RNAi pathway. Thus, it is believed that successful modulation of targets trigger by secreted miRNAs or eTMs, which potentially act as effectors in cross‐talk interaction, can be acquired during successive interaction and improved by evolutionary mechanisms.

## Challenges and future perspectives

Novel MIR gene‐based NBTs have emerged as a powerful strategy to improve several biological or agronomics traits, such as plant tolerance to abiotic and biotic stresses. Although these advances have been remarkable, its advantages and drawbacks need to be discussed critically for the search for best alternatives (Table 2). The constitutive overexpression of specific MIR genes improves agronomic traits in several plant species. However, pleiotropic phenotypes were also observed in these transgenic events in consequence of the wide acting network of the miRNAs. The overexpression driven by tissue‐ or stage‐specific and abiotic and biotic stress‐ or senescence‐inducible promoters are powerful approaches to overcome these undesirable effects (Ferdous *et al*., 2017). The characterization of *cis*‐regulatory elements in promoter sequences of canonical MIR genes can provide knowledge of the transcription factors associated with stress responses and how the MIR genes are regulated by abiotic or biotic stresses. From this, it is possible to better choose the type of promoter to be used, in some cases can opt for the canonical promoter sequence. In addition, artificial MIR genes are a great alternative usually more specific and precise compared to other RNAi strategies in modulation of target mRNA. However, its efficiency is also related to the choice of the backbone and the promoter sequence used for driven its expression. Thus, use of backbone from canonical MIR genes and expression also driven by specific promoters or topical delivery using carrier nanoparticles are some promising alternatives. On the other hand, viral vector‐mediated miRNA delivery may be a usual alternative, but is limited to proofs of concept, since they present some limitations that prevent their use in commercial scale. Unlike the increase in miRNA accumulation, eTM, circRNA and artificial STTM are strategies successfully used to decrease miRNAs cargo in cell and efficiently improve desirable traits. In addition, eTMs modulation showed themselves better than STTM and expression driven by specific promoters can allow its modulation in adequate time and space. Furthermore, prospection of new eTMs or circRNAs, as well as delivery system based on viral vectors, nanoparticles or *Agrobacterium tumefaciens,* provides new alternatives for transgene‐free plants. Genome‐editing technologies have been successfully optimized in plants including modulation of MIR genes and miRNAs. Meganucleases, ZFNs and TALENs although present higher target specificity compared to the new nucleases used in plant genome editing, this high specificity and the limited range of currrenlty available modules restrict its uses to editing few MIR genes. In addition, the assembly of these modules becomes a more expensive and onerous activity compared to the new editing technologies based on CRISPR. The CRISPR/Cas9 NHEJ has successfully allowed the insertion with high specificity of transgene‐free *indels* or knockout gene in several plant genomes. In addition, its high efficiency of editing, high percentage of homozygous mutant already in T0 or T1 generation, easy design and cloning of the binary vector, associated with efficient nanoparticle or biolistic‐mediated CRISPR ribonucleoprotein delivery, provides a powerful alternative for transgene‐free genomic editing. Similarly, CRISPR/Cas9 HDR or HRDR also allows transgene‐free genome editing, deletion of full MIR gene sequence, repair of MIR gene sequence and repair of gene/mRNA becoming resistant to specific miRNAs. However, both HDR and HRDR approaches are limited to low repair or recombination efficiency driven by synthetic donor DNA, added to critical design and onerous construction of binary vector. These drawbacks can be overcome by adjustment of the amount of DNA donor delivered, best selection of totipotent tissues for ribonucleoprotein delivery, use of the new or improved nucleases, and use of transgenic approach with later elimination of the transgene by Mendelian segregation. However, for example, the elimination of the transgene by segregation is not viable in sugarcane due to its vegetative propagation. In addition, efficient transcriptional modulation of MIR genes mediated by CRISPR technology using the deactivated nucleases has allowed to improve agronomic traits. Although it may be a transgenic‐dependent technology with potential of off‐target transcriptional modulation, nucleases expression driven by tissue‐specific or induced promoter, topical delivery of CRISPR ribonucleoprotein and *Agrobacterium tumefaciens*‐mediated transient delivery can overcome these drawbacks. Unlike for these nucleases of DNA, the Cas13a acts with high specificity directly on the RNA molecules. Although it does not yet have many results in plant systems, the current results obtained in mammalian cells are quite promising. In this way, it allows the knock‐down of pre‐, pri‐miRNA, mature miRNA, eTMs, circRNAs and mRNA, both cytoplasmic and nuclear. In addition, Cas13a can be associated with possibility of tissue‐specific and site‐specific RNA editing, transgene‐free approach by topical delivery of CRISPR ribonucleoprotein or Agrobacterium tumefaciens‐mediated transient delivery. Another transgene‐free approach is based on topical delivery of linear or structured pre‐miRNA and mature miRNA. Although not yet fully optimized in plants, its main advantages are the efficient delivery using nanoparticles, high RNA internalization rate in plant cell, low cytotoxic effects and possibility of trans‐kingdom cross‐talk from topical delivery of structured amiRNAs in plants targeting pathogens or insect pest genes. In addition, the possibility of topical delivery of plant miRNAs associated with important traits in crops (acting as enhancers of phenotype or traits). Furthermore, studies on the interkingdom mobility of small RNAs (siRNA or miRNA cross‐talk) may provide evidence to improve the understanding of pathogen–plant, nematode–plant or insect–plant interactions. The elucidation of miRNA cross‐talk in the context of defence response, counter‐defence and plant adaptation based on epigenetic mechanisms can assist in the development of NBTs. Finally, a better understanding of regulation and expression profile that MIR genes associated with abiotic or biotic stress tolerance of the intrinsic features of RNAi machinery in different kingdoms and exploitation of the advantages offered by NBTs may lead to practical biotechnological applications to improve agronomic traits in several crops worldwide. In addition, this knowledge enables the development of new biotechnological products with greater practicality, reduced generation time and low cost.

**Table 2:** Summary of major advantages, drawbacks and alternatives of the new biotechnological tools used to MIR gene or miRNA fine‐tuning

New biotechnological tools	Advantages	Drawbacks	Alternatives
Transgenesis, cisgenesis and intragenesis	Efficient tissue‐specific or induced expression of MIR genes and accumulation of miRNA	Transgenic approach. In consequence of the wide acting network of the miRNAs, its constitutive overexpression often results in pleiotropic effects	Modulation driven by tissue‐, stage‐specific or induced promoters, and canonical promoter sequence
Artificial MIR genes	Modulation of target mRNA is usually more specific compared to RNAi strategies using dsRNA or siRNA	Transgenic approach, backbone selection, potential off‐targets and pleiotropic effects	Use of backbone from canonical MIR genes, expression driven by specific promoters and topical delivery using carrier nanoparticles
Viral vector‐mediated miRNA delivery	Transgene‐free approach and suitable for proofs of concept	Restricted host plant range, adult plant resistance, very limited to nucleic acids length and viral infection usually restricted to young tissue or meristems	Development of new and optimized viral vectors
Endogenous (eTM), circular (circRNAs) and artificial short tandem target mimicry (STTM)	Efficient sequestration of miRNAs, eTMs show themselves better than STTM, high stability of circRNAs and expression driven by specific promoters allows modulation in adequate time and space	Transgenic approach and backbone selection	Prospection and characterization of new miRNA‐specific eTMs, viral vector‐ or nanoparticle‐mediated eTMs or STMM delivery, and *Agrobacterium tumefaciens*‐mediated transient delivery
Meganucleases, zinc‐finger proteins and TALEN	Greater target specificity and allows target any DNA sequence	Off‐target cleavage, high specificity of the Meganucleases and limited range of ZFN and TALEN modules restrict its use to editing few MIR genes. More onerous and expensive compared to CRISPR system	Use of new genome‐editing technologies
CRISPR/Cas9 or/Cpf1 non‐homologous end joining (NHEJ)	Transgene‐free *indels* or knockout, biolistic‐mediated CRISPR ribonucleoprotein delivery, target any DNA sequence, highly efficient editing, high percentage of homozygous mutant already in T0 or T1 generation, easy design and cloning of the binary vector	Off‐target cleavage and selection of target site is limited by requirement of PAM motif adjacent	Use of new or improved nucleases
CRISPR/Cas9 or/Cpf1 homology‐directed repair (HDR) or homology and recombination‐directed repair (HRDR)	Biolistic‐mediated CRISPR ribonucleoprotein delivery, target any DNA sequence, transgene‐free genome editing, deletion of full MIR gene sequence, repair of MIR gene sequence, repair of gene/mRNA becoming resistant to specific miRNAs	Low repair efficiency driven by donor DNA or low recombination efficiency, critical design and onerous construction of binary vector	Biolistic‐mediated CRISPR ribonucleoprotein delivery, adjustment of the amount of DNA donor delivery, use of new or improved nucleases, use of transgenic approach with elimination of transgene by Mendelian segregation
CRISPR/dCas9 or/dCpf1	Efficient transcriptional modulation of endogenous genes associated to important traits, expression driven by specific promoters and epigenome editing	Transgenic approach and off‐target transcriptional modulation	Nucleases expression driven by tissue‐specific or induced promoter, topical delivery of CRISPR ribonucleoprotein and *Agrobacterium tumefaciens*‐mediated transient delivery
CRISPR/Cas13a, Cas13b or dCas13	High specificity of target RNA, greater knock‐down efficiency of pre‐, pri‐miRNA, mature miRNA, eTMs and mRNA, both cytoplasmic or nuclear and possibility of tissue‐specific and site‐specific RNA editing	Technology still being established in plants, may require transgenic approaches depending on the strategy used and transient editing	Linking previous findings in mammalian cells with proofs of concept in plants, topical delivery of CRISPR ribonucleoprotein and *Agrobacterium tumefaciens*‐mediated transient delivery
Topical delivery of linear or structured pre‐miRNA and mature miRNA	Transgene‐free approach, efficient delivery using nanoparticles, high RNA internalization rate in plant cell, low cytotoxic effects, trans‐kingdom cross‐talk from topical delivery of structured amiRNAs in plants targeting pathogens or insect pest, and topical delivery of plant miRNAs associated to important traits	Delivery system and nanoparticle not yet fully optimized for plants, high cost for production of RNA in large‐scale potential of off‐targets modulation	Proofs of concept in model or crop plants and process optimization of dsRNA production

## Conflict of interest statement

The authors declare that the research was conducted in the absence of any commercial or financial relationships that could be construed as a potential conflict of interest.

## Author contributions

MFB wrote the manuscript; PCGF, AKK, FGH, ALN, HBCM and MFGS revised and provided inputs. All authors read and approved the final version.
